# Chronic thoracic spinal cord injury impairs CD8^+^ T-cell function by up-regulating programmed cell death-1 expression

**DOI:** 10.1186/1742-2094-11-65

**Published:** 2014-04-01

**Authors:** Ji Zha, Annalise Smith, Samita Andreansky, Valerie Bracchi-Ricard, John R Bethea

**Affiliations:** 1 The Miami Project to Cure Paralysis, Department of Neurosurgery, Miller School of Medicine, University of Miami, Miami, FL 33136, USA; 2 Department of Microbiology and Immunology, Miller School of Medicine, University of Miami, Miami, FL 33136, USA; 3 Department of Pediatrics and Medicine, Miller School of Medicine, University of Miami, Miami, FL 33136, USA; 4 Department of Biology, Drexel University, Philadelphia, PA 19104, USA

**Keywords:** Spinal cord injury, T-cell exhaustion, PD-1, Norepinephrine

## Abstract

**Background:**

Chronic spinal cord injury (SCI) induces immune depression in patients, which contributes to their higher risk of developing infections. While defects in humoral immunity have been reported, complications in T-cell immunity during the chronic phase of SCI have not yet been explored.

**Methods:**

To assess the impact of chronic SCI on peripheral T-cell number and function we used a mouse model of severe spinal cord contusion at thoracic level T9 and performed flow cytometry analysis on the spleen for T-cell markers along with intracellular cytokine staining. Furthermore we identified alterations in sympathetic activity in the spleen of chronic SCI mice by measuring splenic levels of tyrosine hydroxylase (TH) and norepinephrine (NE). To gain insight into the neurogenic mechanism leading to T-cell dysfunction we performed *in vitro* NE stimulation of T-cells followed by flow cytometry analysis for T-cell exhaustion marker.

**Results:**

Chronic SCI impaired both CD4^+^ and CD8^+^ T-cell cytokine production. The observed T-cell dysfunction correlated with increased expression of programmed cell death 1 (PD-1) exhaustion marker on these cells. Blocking PD-1 signaling *in vitro* restored the CD8^+^ T-cell functional defect. In addition, we showed that chronic SCI mice had higher levels of splenic NE, which contributed to the T-cell exhaustion phenotype, as PD-1 expression on both CD4^+^ and CD8^+^ T-cells was up-regulated following sustained exposure to NE *in vitro*.

**Conclusions:**

These studies indicate that alteration of sympathetic activity following chronic SCI induces CD8^+^ T-cell exhaustion, which in turn impairs T-cell function and contributes to immune depression. Inhibition of the exhaustion pathway should be considered as a new therapeutic strategy for chronic SCI-induced immune depression.

## Background

Spinal cord injury (SCI) is a devastating condition that affects more than 200,000 people in the United States. Besides the obvious locomotor impairments, SCI brings about a wide array of metabolic and immune problems for those patients, especially now that their life expectancy has considerably increased [1]. Patients suffering from SCI are at higher risk than the general population of developing infections and their prognosis is often much poorer [2-4]. Originally the immunodeficiency syndrome observed in SCI patients was thought to be due for the most part to high levels of glucocorticoids. However, the extent of immune depression is much more pronounced with injuries to the central nervous system than with any other stressful traumatic events suggesting a neurogenic component to the immunodeficiency [5,6]. Over the past decade it became clear that the immune system is in close relationship with the nervous system and that neuro-immune communications are essential to develop an appropriate immune response to pathogens [7,8].

The immune organs are highly innervated by the sympathetic nervous system (SNS) [9], and through the release of norepinephrine (NE), the SNS has been reported to regulate the functions of both innate and adaptive immune cells [10]. Tracing studies using pseudorabies virus injection in the spleen labeled the thoracic spinal cord segments from level 3 to level 12 [11]. Thus, traumatic injury to the thoracic spinal cord may disrupt the sympathetic preganglionic neurons at the injury level and in turn may alter the sympathetic innervation of the spleen.

Recent studies using rodent SCI models have begun to investigate the effect of chronic SCI (> four weeks) on the adaptive immunity. Chronic SCI impairs the antibody response in both mice and rats [12-14]. The germinal center B-cells’ number and function are also impaired by chronic SCI [13]. Importantly, T-cells have a key role in both antibody mediated and cytotoxic immune response to viral infections such as influenza [15]. It has been shown that CD4^+^ T-cell effector function to mouse hepatitis virus (MHV) is suppressed following SCI in the acute phase [16]. However, whether or not SCI alters T-cell function over a chronic time period has yet to be explored.

T-cell exhaustion and its correlation with immune defects have been widely studied in chronic viral infection, aging and cancer research in the last decade [17-23]. Elevated expression of exhaustion markers such as programmed cell death-1 (PD-1) negatively regulates T-cell effector function. Blockade of exhaustion marker pathways has been shown to restore the T-cell functional defects in both chronic virus infection and tumor models [18,24-28]. Higher viral load, persistent exposure to antigen and loss of CD4^+^ T-cell correlates with T-cell exhaustion [29]. It is unclear whether T-cell exhaustion is responsible for chronic SCI-induced immune depression and whether change in SNS activity affects T-cell exhaustion.

In the present study, we investigated the impact of chronic SCI on the peripheral T-cell immunity. We provide evidence that cytokine production by CD4^+^ and CD8^+^ T-cells from chronically injured mice is impaired and that this impairment was due to increased expression of PD-1 exhaustion marker on splenic T-cells. Blocking PD-1 rescued the functional defects of T-cells isolated from chronic SCI mice. We also demonstrated that increased levels of splenic NE following SCI may contribute to increased PD-1 expression on T-cells as we showed *in vitro* that PD-1 expression is increased on T-cells in presence of sustained levels of NE. Collectively, these findings suggest that deregulation of splenic sympathetic activity by chronic SCI induces T-cell exhaustion, which in turn results in T-cell dysfunction and immune depression.

## Methods

### Animals

Age-matched female C57BL/6 mice were purchased from The Jackson Laboratory or bred in the Animal Facility of the Miami Project to Cure Paralysis. All mice used for the experiments were four to seven months old when sacrificed. All animal protocols were approved by the University of Miami Institutional Animal Care and Use Committee (IACUC) and are in accordance with National Research Council guidelines for the care and use of laboratory animals.

### Spinal cord injury

Severe spinal contusion injury was induced using the Infinite Horizon Impactor (Precision Systems and Instrumentation, LLC). Briefly, three to four month-old mice (weight ± SD: 19.9 ± 1.5 g) were acclimated for one week prior to surgery. Mice were anesthetized by intraperitoneal injection of ketamine (100 mg/kg) and xylazine (10 mg/kg). A laminectomy was performed at vertebrae thoracic level 9 (T9). The underlying spinal cord was exposed and injured by the tip of the contusion device at a predetermined impact force of 70 kDynes (severe injury). After surgery, mice were housed separately and received daily subcutaneous injections of lactated Ringer’s solution to prevent fluid loss and gentamicin (40 mg/kg) to prevent urinary tract infections. Manual bladder expression (twice daily) was performed until mice regain bladder function. After about three weeks mice were reunited with their original cage mates.

### Splenocyte isolation

Mice were anesthetized and a laparotomy was performed to expose and excise the spleen. Single cell suspensions of individual spleens were prepared by mashing the spleens through a 100-μm nylon mesh strainer. Strainers were washed with Hank’s Balanced Salt Solution (HBSS, Gibco). Red blood cells were lysed with ACK lysing buffer (Gibco, Grand Island, NY). For flow cytometry staining, splenocytes were washed with HBSS, resuspended in flow cytometry (FACS) staining buffer (HBSS, 1% BSA, 0.05% sodium azide). For *ex vivo* stimulation assay, splenocytes were washed with complete RPMI (RPMI 1640, 5% FBS, 100 U/mL penicillin, 100 μg/mL streptomycin). The number of live cells was determined by trypan blue exclusion staining.

### Flow cytometry

Prior to staining, all samples were incubated with 5 μg/mL Fc receptor block (anti-mouse CD16/32, Biolegend, San Diego, CA.) for five minutes on ice to prevent nonspecific staining. Cells were stained for surface markers by adding the following conjugated Abs: APC-anti-CD11c (Biolegend, San Diego, CA. clone N418, 1:100), PE-anti-CD274 (Biolegend, San Diego, CA. PD-L1, clone 10F.9G2, 1:100), APC/Cy7-anti-CD4 (Biolegend, San Diego, CA. clone GK1.5, 1:200), Alexa Fluor 488-anti-CD8a (Biolegend, San Diego, CA. clone 53-6.7, 1:200) and PE/Cy7-anti-CD279 (Biolegend, San Diego, CA. PD-1, clone 29F.1A12, 1:200), APC-efluor780-anti-B220 (eBioscience, San Diego, CA. clone HIS24, 1:200), PE-Cy7-anti-CD11b (eBioscience, San Diego, CA. clone M1/70, 1:200), PE/Cy7-anti-CD45 (eBioscience, San Diego, CA. clone 30-F11, 1:10,000), FITC-anti-CD45 (eBioscience, San Diego, CA. clone 30-F11, 1:200), Alexa Fluor 488-anti-CD3e (eBioscience, San Diego, CA. clone 145-2C11, 1:200), efluor450-anti-CD3 (eBioscience, San Diego, CA. clone 17A2, 1:200), APC-anti-CD4 (eBioscience, San Diego, CA. 1:100), PE-anti-CD4 (eBioscience, San Diego, CA. clone GK1.5, 1:200), APC-anti-CD8a (eBioscience, San Diego, CA. clone 53-6.7, 1:200), PE-anti-CD8a (eBioscience, San Diego, CA. clone 53-6.7, 1:100). For surface antibody staining, cells were then fixed overnight with FACS buffer containing 1% paraformaldehyde and resuspended in FACS buffer. For detection of dead/live cells, unfixed cells were incubated with 5 μL of 7-AAD Viability Staining Solution (Biolegend, San Diego, CA) and immediately analyzed by flow cytometry. For intracellular antibody staining, cells are fixed, permeablized and stained using Foxp3 staining Kit (eBiosicence, San Diego, CA.) according to the manufacture’s protocol. Intracellular marker expression was quantified using the following conjugated Abs: eFluor450-anti-IFN-γ (eBioscience, San Diego, CA. 1:100) and PerCP-eFluor 710-anti-TNFα (eBioscience, San Diego, CA. 1:100). All antibody incubations were performed for 20 minutes at 4°C. The following isotype control antibodies were used for flow cytometry gating: PerCP-eFluor710 IgG1κ (clone eBRG1, eBioscience, San Diego, CA), eFluor450 IgG1κ (clone eBRG1, eBioscience, San Diego, CA), APC/Cy7 IgG2b κ (clone RTK4530, Biolegend, San Diego, CA) and APC IgG2a κ (clone eBR2a, eBioscience, San Diego, CA). Cells were analyzed using BD LSRII, BD LSR Fortessa-HTS or BD FACS. Canto-II flow cytometers and were quantified using FACS-Diva Version 6.1.3 software (BD Biosciences, San Jose, CA). Gating strategies were described in Additional files 1 and 2.

### T-cell stimulation

Splenocytes were isolated and counted as above. For PMA/ionomycin stimulation, 10^6^ splenocytes were cultured in 1 mL of complete RPMI in a 24-well plate with 50 ng/mL phorbol myristate acetate (PMA, Sigma, St. Louis, MO), 0.75 μg/mL ionomycin calcium salt (Sigma, St. Louis, MO) and 1 μL GolgiPlug protein transport inhibitor (BD Biosciences, San Jose, CA) for four hours at 37°C, 5% CO_2_. For the PD-1 blockade assay, 10 μg/mL anti-PD-1 blocking antibody (Biolegend, San Diego, CA) or 10 μg/mL rat IgG2a, κ isotype control (Biolegend, San Diego, CA) was added to the complete RPMI along with PMA and ionomycin. For the T-cell receptor (TCR) activation assay, 96-well plates were coated with 10 μg/mL anti-mouse CD3e (eBioscience, San Diego, CA. clone 145-2C11) overnight at 4°C. The next day splenocytes (10^6^/well) were added in 200 μL T-cell medium (RPMI, 10% FBS, 55 μM 2-mercaptoethanol, 2 mM L-glutamine, 10 μg/ml gentamicin and 10 μM HEPES) along with 2 μg/mL of anti-mouse CD28 (eBioscience, San Diego, CA. clone 37.51) and 50 U/mL of recombinant human IL-2 (Peprotech, Rocky Hill, NJ) and cultured for three days at 37°C in 5% CO_2_. Brefeldin A (eBioscience, San Diego, CA) was added six hours before intracellular cytokine cell staining.

### ELISA measurement of cytokine concentration

Supernatants from T-cell stimulation (three-days of TCR activation as described above) were collected and frozen at −80°C. TNF-α and IFN-γ protein levels were measured using the Mouse TNF-α ELISA Ready-SET-Go! Kit (eBioscience, San Diego, CA) and the Mouse IFN- γ ELISA Ready-SET-Go! Kit (eBioscience, San Diego, CA), respectively, according to the manufacture’s protocol. For TNF-α, supernatants were diluted 1:5 and for IFN-γ 1:50.

### T-cell proliferation assay

Isolated splenocytes from CT and chronic SCI mice were resuspended in 1 ml PBS containing 5% FBS. Immediately after addition of carboxyfluorescein diacetate succinimidyl ester (CFSE, 5 μM) cells were mixed thoroughly and incubated for five minutes at room temperature. Following incubation, free CFSE was quenched with 10 ml T-cell medium (RPMI, 10% FBS, 2-mercaptoethanol (55 μM), l-glutamine (2 mM), gentamicin (10 μg/ml) and HEPES (10 μM)). After extensive washes in T-cell medium, cells were diluted to 1 × 10^6^/ml and seeded in an anti-CD3e-coated 96-well plate at 200 μl/well. Anti-mouse CD28 (eBioscience, San Diego, CA, clone 37.51, 2 μg/mL) and 10 U/mL of recombinant human IL-2 (Peprotech, Rocky Hill, NJ) were also added to these wells for further stimulation. Cells were subsequently allowed to proliferate at 37°C in 5% CO_2_ for three days before staining using APC anti-CD4 and PE anti-CD8. Flow cytometry data (20,000 events gated on CD4 or CD8) were acquired on LSR II flow cytometer (BD Biosciences, San Jose, CA). Data analysis was performed using Flowjo Software (TreeStar Inc, Ashland, OR).

### T-cell enrichment

T-cell enrichment was obtained by plating splenocytes in a 75 cm^2^ cell culture flask coated with 100 μg/mL goat anti-mouse IgG + IgM (H + L) (Jackson Immuno Research Laboratories, West Grove, PA) for one hour, followed by re-plating the non-adherent cells in another 75 cm^2^ cell culture flask for one hour at 37°C, 5% CO_2_. The non-adherent T-cells were then collected and washed with complete RPMI before used in stimulation assays.

### Norepinephrine and T-cell exhaustion

To measure the effects of NE on T-cell exhaustion, T-cells from uninjured (CT) mice were enriched and counted as described above. Prior to cell culture, a fresh stock solution of 100 mM NE was prepared by dissolving 50 mg of (−)-norepinephrine (Sigma, St. Louis, MO) in 2.96 mL of 0.4 N perchloric acid (PCA) containing 5 mM glutathione. Enriched T-cells (2 × 10^5^) were cultured in 200 μL of cRPMI with either NE diluted to a final concentration of 10 μM or an equivalent volume of perchloric acid/glutathione solution as vehicle control. Cells were cultured for one day, two days or three days at 37°C, 5% CO_2_ and harvested for flow cytometry analysis. Culture medium with NE or vehicle was changed daily. To analyze T-cell cytokine production following NE exposure, we replaced the media with cRPMI in a 24-well plate with 50 ng/mL phorbol myristate acetate (PMA, Sigma, St. Louis, MO), 0.75 μg/mL ionomycin calcium salt (Sigma, St. Louis, MO) and brefeldin A solution (eBioscience, San Diego, CA) for four hours at 37°C, 5% CO_2_.

### Protein extraction and Western blotting

Spleens were harvested and homogenized in radio-immunoprecipitation assay lysis buffer (10 mM Na-phosphate pH 7.2, 150 mM NaCl, 1% Igepal CA-630, 1% Na-deoxycholate, 0.1% sodium dodecyl sulfate, 2 mM ethylenediaminetetraacetic acid) containing complete protease inhibitor cocktail (Roche Diagnostics, Indianapolis, IN) and phosphatase inhibitor cocktail 3 (Sigma, St. Louis, MO). The samples were mixed end-over-end at 4°C for 20 minutes and lysates were centrifuged at 4°C for 15 minutes at 14,000 rpm. The protein concentrations in the supernatant were quantified using DC Protein Assay Kit (Bio-Rad, Hercules, CA). Protein samples (20 μg) and protein prestained standards (Precision Plus, Sanborn, NY) were resolved on a 10% sodium dodecyl sulfate polyacrylamide gel electrophores (SDS-PAGE) and then transferred to nitrocellulose membrane (Bio-Rad, Hercules, CA). Membranes were blocked for one hour at room temperature in Tris-buffered saline with Tween-20 (TBST) containing 5% non-fat dry milk followed by incubation overnight at 4°C with rabbit anti-tyrosine hydroxylase antibody (1:1,000, Calbiochem, San Diego, CA). After extensive washes in TBST, a conjugated horseradish peroxidase anti-rabbit secondary antibody was then applied for 30 minutes at room temperature. Immunoreactive signals were visualized using enhanced chemiluminescence (ECL) Western blotting detection reagents (Amersham, Piscataway, NJ). The protein bands were quantified with Quantity One software (Bio-Rad, Hercules, CA) and normalized to β-actin.

### Measurement of splenic norepinephrine

Spleens were snap frozen in liquid nitrogen and ground to powder taking care to avoiding any thawing. Glutathione/PCA solution (0.4 N perchloric acid with 5 mM glutathione) was added to the powder at 1 mL per 100 mg of tissue. Following homogenization, the samples were centrifuged for 15 minutes at 14,000 rpm and 4°C and the supernatants were stored at −80°C. Concentrations of NE in the supernatant were measured by the Hormone Assay & Analytical Services Core at Vanderbilt University School of Medicine.

### Data analysis

All experimental data are expressed as mean ± standard error of the mean (SEM). Student’s *t*-test was applied for comparison between two groups. Graphpad Prism (Graphpad Software, La Jolla, CA, USA) was used for statistical analysis. *P* < 0.05 was considered as significant.

## Results

### Chronic SCI alters the function of CD4^+^ and CD8^+^ T-cells but not their numbers

**Figure 1:**
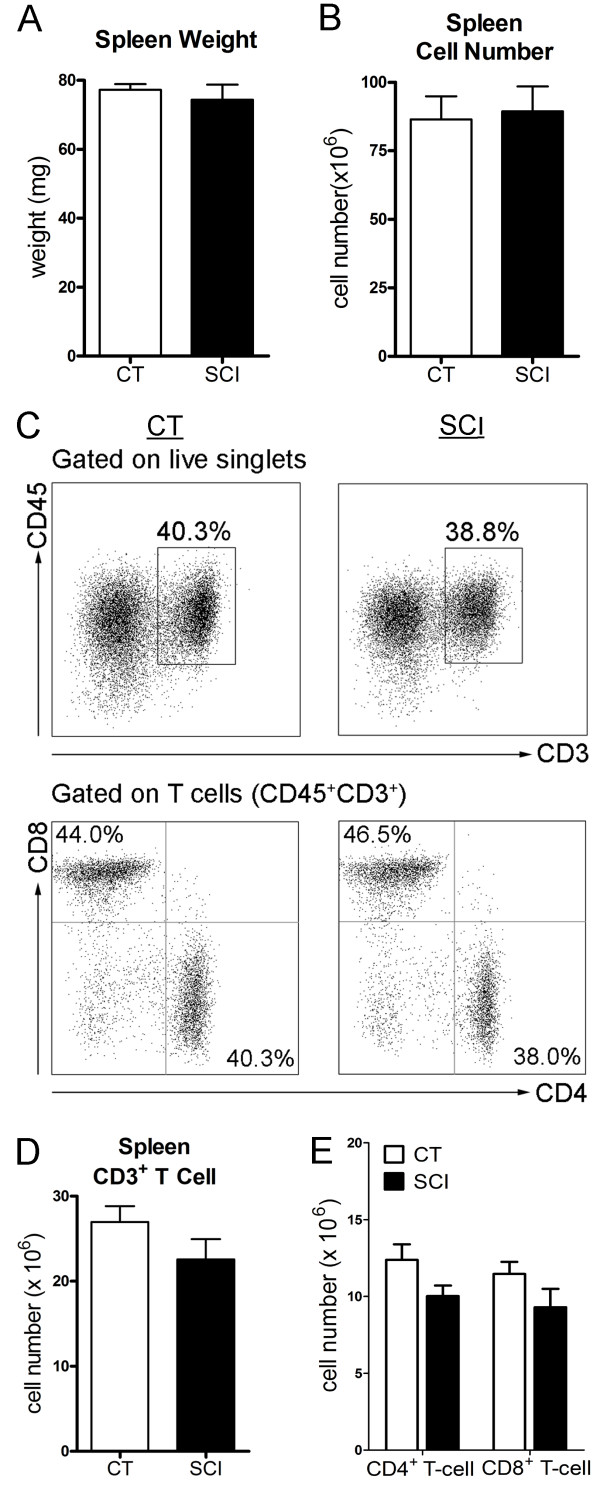
**The number of splenocytes and splenic T-cells are not changed during chronic spinal cord injury (SCI). (A)** Bar graph represents the mean ± SEM spleen weights of uninjured mice (CT) and T9-SCI mice at chronic phase after injury (SCI). n = 12 mice/group. Data are pooled across three independent experiments. **(B)** Bar graph represents the mean ± SEM of total splenocyte numbers for CT and SCI mice. n = 17 for CT, n = 19 for SCI. Data were pooled across four independent experiments. **(C)** Representative dot plots show the percentage of T-cells (CD45^+^CD3^+^) in live splenocytes (upper panels), as well as the percentages of CD4^+^ T-cells (CD4^+^CD8^−^, bottom right quadrant) and CD8^+^ T-cells (CD4^−^CD8^+^, upper left quadrant) in gated T-cells (bottom panels). **(D)** Bar graph represents the mean ± SEM number of splenic T-cells in CT and SCI mice. **(E)** Bar graph show the mean ± SEM numbers of splenic CD4^+^ T-cells and CD8^+^ T-cells in CT and SCI mice. Ten thousand events gated on live singlets were collected. n = 12 mice/group. Data are pooled across three independent experiments. No statistical difference was detected between the two groups. *P* > 0.05, two-tailed Student’s *t*-test.

Several studies have shown that following acute SCI (< seven days post-injury) there was a dramatic reduction in the number of splenic T-cells [30-32]. To evaluate the impact of spinal cord injury on spleen function at a chronic time point, namely five to seven weeks post-SCI, we first assessed the cell numbers with a focus on T-cells. As shown in Figure 1A, the spleen weight was not significantly different between the uninjured and chronic SCI groups (uninjured: 77.1 ± 2.1 mg; chronic SCI: 70.6 ± 5.3 mg; *P* = 0.27) nor was the total spleen cell number (uninjured: 86.5 ± 8.4 × 10^6^; chronic SCI: 89.4 ± 9.2 × 10^6^; *P* = 0.82) (Figure 1B). Furthermore, the spleens of uninjured and chronic SCI mice contained similar numbers of T-cells (uninjured: 27.0 ± 1.9 × 10^6^; chronic SCI: 22.6 ± 2.4 × 10^6^; *P* = 0.16) (Figure 1C, D) with no significant differences in the numbers of CD4^+^ T-cells (uninjured: 12.4 ± 1.0 × 10^6^; chronic SCI: 10.0 ± 0.7 × 10^6^; *P* = 0.06) or CD8^+^ T-cells (uninjured: 11.5 ± 0.8 × 10^6^; chronic SCI: 9.3 ± 1.2 × 10^6^; *P* = 0.14) (Figure 1C, E) between groups.

**Figure 2:**
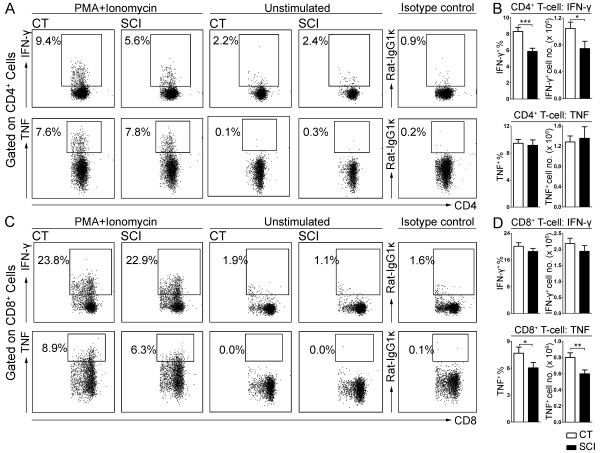
**Impaired T-cell cytokine production in response to PMA/ionomycin stimulation after chronic spinal cord injury (SCI).** Isolated splenocytes (1 × 10^6^) from uninjured (CT) or T9-SCI mice at chronic phase after injury (SCI) were stimulated *ex vivo* with PMA/ionomycin in the presence of brefeldin A for four hours and then processed for flow cytometry analysis. The unstimulated controls were incubated only with brefeldin A. **(A)** Representative dot plots show the percentage of IFN-γ^+^ and TNF-α^+^ cells in gated CD4^+^ T-cells following PMA/ionomycin stimulation compared to unstimulated or isotype control. **(B)** Bar graph represents the mean ± SEM percentages and numbers of cytokine producing CD4^+^ T-cell in response to PMA/ionomycin stimulation. **(C)** Representative dot plots show the percentage of IFN-γ^+^ and TNF-α^+^ cells in gated CD8^+^ T-cells following PMA/ionomycin stimulation compared to unstimulated or isotype control. **(D)** Bar graph represents the mean ± SEM percentages and numbers of cytokine producing CD8^+^ T-cells in response to PMA/ionomycin stimulation. Ten thousand events gated on live singlets were collected. n = 14 for CT, n = 12 for SCI. Data were pooled across four independent experiments. *P < 0.05, **P < 0.01, ***P < 0.001, one-tailed Student’s *t*-test.

Effective T-cell response against pathogen requires the production of cytokines such as IFN-γ and TNF-α [33-36]. To evaluate the function of these T-cells, we isolated splenocytes from the spleens of uninjured and chronic SCI mice and stimulated them *ex vivo* with PMA/ionomycin. As shown in Figure 2A and 2B, a significantly smaller percentage of CD4^+^ T-cells produced IFN-γ following stimulation (uninjured: 8.3 ± 0.5%; chronic SCI: 5.8 ± 0.4%; *P* = 0.0005) also correlating with a significantly reduced number of IFN-γ^+^CD4^+^ T-cells in chronically injured mice compared to controls (uninjured: 1.0 ± 0.1 × 10^6^; chronic SCI: 0.7 ± 0.1 × 10^6^; *P* = 0.02). The percentage or number of CD4^+^ T-cell expressing TNF-α (Figure 2A, B) following stimulation was not significantly different between chronic SCI and control (percentage: uninjured: 9.5 ± 0.6%; chronic SCI: 9.1 ± 0.8%; *P* = 0.37; cell number: uninjured: 1.3 ± 0.1 × 10^6^; chronic SCI: 1.4 ± 0.2 × 10^6^; *P* = 0.38). However, the percentage and number of CD8^+^ T-cells expressing TNF-α was significantly reduced following chronic SCI (percentage: uninjured: 7.7 ± 0.7%; chronic SCI: 6.0 ± 0.6%; *P* = 0.04; cell number: uninjured: 0.80 ± 0.06 × 10^6^; chronic SCI: 0.60 ± 0.05 × 10^6^; *P* = 0.007) (Figure 2C, D), when neither the percentage nor the number of IFN-γ^+^CD8^+^ T-cells was changed by chronic SCI (percentage: uninjured: 20.1 ± 1.1%; chronic SCI: 18.5 ± 0.9%; *P* = 0.14; cell number: uninjured: 2.2 ± 0.2 × 10^6^; chronic SCI: 1.9 ± 0.2 × 10^6^; *P* = 0.17) (Figure 2C, D).

**Figure 3:**
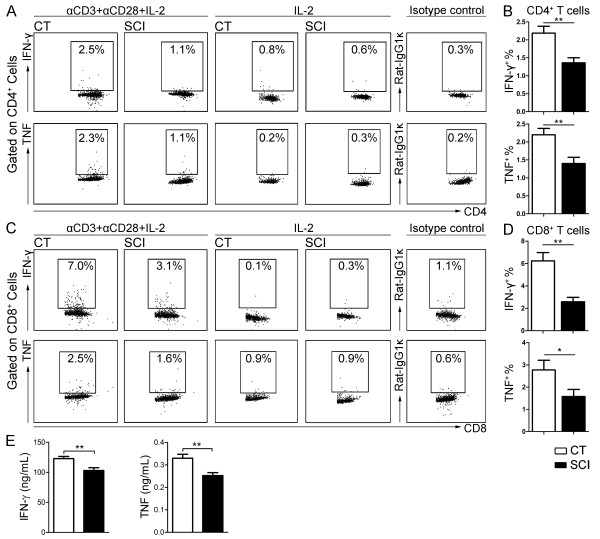
**Impaired T-cell cytokine production following T-cell receptor (TCR) activation in chronic spinal cord injury (SCI) mice.** Isolated splenocytes (1 × 10^6^) from uninjured (CT) or T9-SCI mice at chronic phase after injury (SCI) were stimulated *ex vivo* for three days with anti-CD3 + anti-CD28 + IL-2 or with IL-2 only. Brefeldin A was added six hours before cell collection. Intracellular cytokine staining and flow cytometry analysis were performed. **(A)** Representative dot plots show the percentage of IFN-γ^+^ cells and TNF-α^+^ cells in gated CD4^+^ T-cells following three-day stimulation with anti-CD3 + anti-CD28 + IL-2 or with IL-2 only. **(B)** Bar graph represents the mean ± SEM percentages of cytokine producing CD4^+^ T-cell in response to TCR activation. **(C)** Representative dot plots show the percentage of IFN-γ^+^ cells and TNF-α^+^ cells in gated CD8^+^ T-cells following three-day stimulation with anti-CD3 + anti-CD28 + IL-2 or with IL-2 only. **(D)** Bar graph represents the mean ± SEM percentages of cytokine producing CD8^+^ T-cells in response to TCR activation. n = 4 for CT, n = 5 for SCI. Twenty thousand events gated on live singlets were collected for flow cytometry analysis. n = 5 mice per group. *P < 0.05, **P < 0.01, one-tailed Student’s *t*-test. **(E)** The concentration of IFN-γ and TNF-α in the supernatant of stimulated cells was measured by ELISA.

To confirm the functional impairment of T-cell cytokine production isolated from chronically injured mice, we repeated the experiment using anti-CD3/anti-CD28 stimulation protocol which is more physiologically relevant. As shown in Figure 3A, B, the percentages of CD4^+^ T-cells producing IFN-γ or TNF-α following anti-CD3/anti-CD28 stimulation were significantly decreased in the SCI group (IFN-γ: uninjured: 2.2 ± 0.2%; chronic SCI: 1.4 ± 0.1%; *P* = 0.005; TNF-α: uninjured: 2.2 ± 0.2 × 10^6^; chronic SCI: 1.4 ± 0.2 × 10^6^; *P* = 0.008). CD8^+^ T-cells from chronic SCI mice also show a reduction in the percentages of IFN-γ and TNF-α expressing cells in response to anti-CD3/anti-CD28 stimulation (IFN-γ: uninjured: 6.2 ± 0.7%; chronic SCI: 2.6 ± 0.4%; *P* = 0.001; TNF-α: uninjured: 2.8 ± 0.4 × 10^6^; chronic SCI: 1.6 ± 0.3 × 10^6^; *P* = 0.03) Figure 3C, D. To further confirm the deficiency in cytokine production, we performed ELISA on the supernatant of these stimulated T-cells and found a significant reduction in the concentration of IFN-γ (uninjured: 123 ± 4 ng/ml; chronic SCI: 103 ± 5 ng/ml; *P* = 0.005) and TNF-α (uninjured: 0.33 ± 0.02 ng/ml; chronic SCI: 0.25 ± 0.01 ng/ml; *P* = 0.004) (Figure 3E).

**Figure 4:**
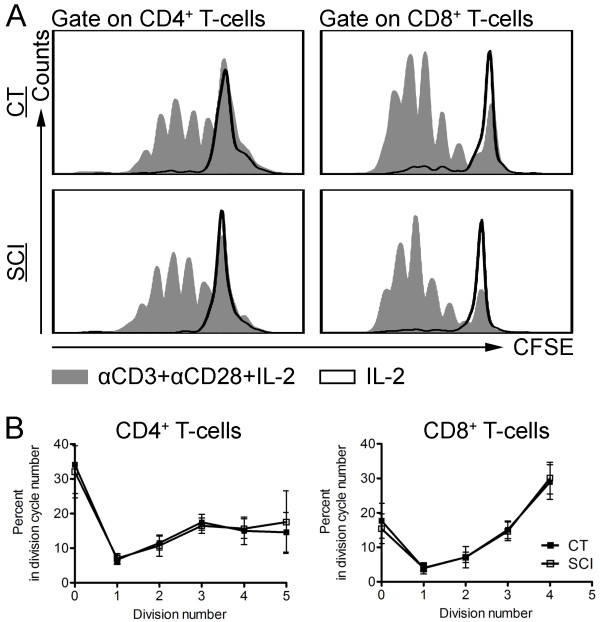
**T-cells *in vitro *proliferation shows no change in the mice with chronic spinal cord injury (SCI).** Isolated splenocytes (2 × 10^5^) from uninjured (CT) or T9-SCI mice at chronic phase after injury (SCI) were labeled with CFSE and stimulated *ex vivo* for three days with anti-CD3+CD28+IL-2 or with IL-2 only. **(A)** Representative histogram plots show the CFSE fluorescence of CD4^+^ T-cells and CD8^+^ T-cells following three-day stimulation with anti-CD3+CD28+IL-2 or with IL-2 only. **(B)** The line graph shows the percentage of CD4^+^ T-cells and CD8^+^ T-cells that have undergone zero to five divisions. Twenty thousand events gated on CD4^+^ T-cells or CD8^+^ T-cells were collected. n = 5 mice per group. No statistical difference was detected between the two groups. *P* > 0.05, two-tailed Student’s *t*-tests were performed for each cell division number.

Since stimulation with anti-CD3/anti-CD28 in presence of IL-2 induces proliferation of T-cells, we also evaluated the proliferative capabilities of these cells isolated from chronically injured mice using CFSE (Figure 4A, B). We did not find any significant difference in the ability of either CD4^+^ T-cells or CD8^+^ T-cells to proliferate compared to CT mice.

Collectively, we showed that both CD4^+^and CD8^+^ T-cells isolated from chronic SCI mice have defects in cytokine production, which may contribute to the chronic SCI induced immunodeficiency.

### Increased expression of exhaustion marker PD-1 on T-cells isolated from chronic SCI mice

**Figure 5:**
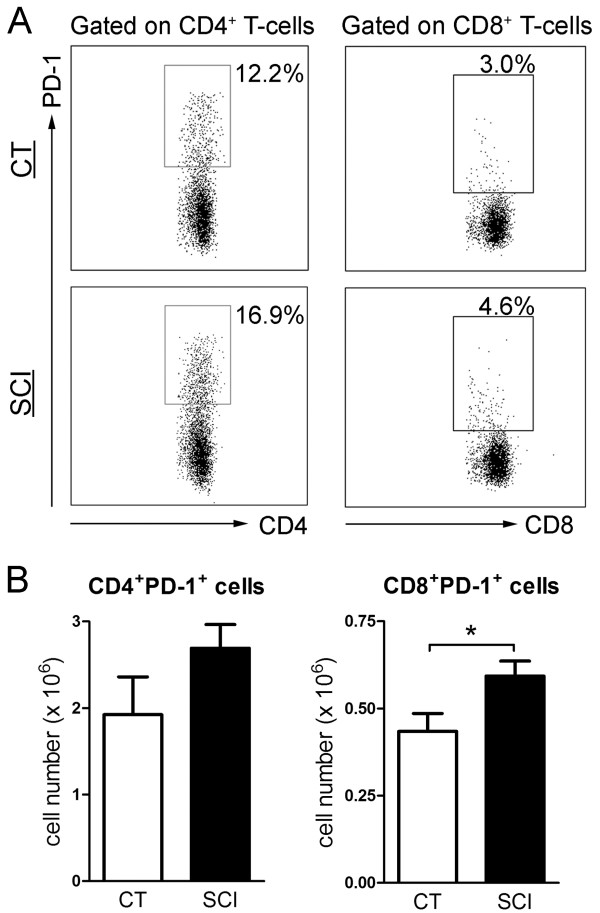
**Increased expression of exhaustion marker PD-1 on T-cells isolated from chronic spinal cord injury (SCI) mice. (A)** Representative dot plots show the percentage of PD-1^+^ cells in gated CD4^+^ T-cells and CD8^+^ T-cells from uninjured (CT) and T9-SCI mice at chronic phase after injury (SCI). **(B)** Bar graphs show the mean ± SEM numbers of PD-1 expressing CD4^+^ T-cells and CD8^+^ T-cells. Twenty thousand events gated on live singlets were collected. n = 9 for CT mice, n = 11 for SCI mice. Data have been pooled across two independent experiments. *P < 0.05, one-tailed Student’s *t*-test.

T-cell exhaustion indicated by increased expression of exhaustion markers such as PD-1, cytotoxic T-lymphocyte antigen 4 (CTLA-4), T-cell immunoglobulin mucin-3 (TIM-3) and lymphocyte activation gene-3 (LAG-3) was shown to correlate with T-cell dysfunction in chronic viral infection models and aging animals [23,25,29,37-40]. To examine whether the T-cell impairment in cytokine production observed in chronic SCI mice was associated with T-cell exhaustion, we measured the expression of exhaustion markers on both CD4^+^ T-cells and CD8^+^ T-cells. The percentage of PD-1 expressing cells in both CD4^+^ T-cells and CD8^+^ T-cells was significantly higher in the spleen of chronic SCI mice compared with uninjured controls (CD4^+^PD1^+^ T-cells: uninjured: 12.9 ± 1.3%; chronic SCI: 18.0 ± 1.7%; *P* = 0.02. CD8^+^PD1^+^ T-cells: uninjured: 3.4 ± 0.2%; chronic SCI: 5.0 ± 0.4%; *P* = 0.003) (Figure 5A). The number of splenic CD8^+^PD-1^+^ cells was also significantly increased in the chronic SCI mice (uninjured: 0.43 ± 0.05 × 10^6^; chronic SCI: 0.59 ± 0.04 × 10^6^; *P* = 0.01) (Figure 5B). However, the number of splenic CD4^+^PD-1^+^ cells was not significantly changed by chronic SCI (uninjured: 1.9 ± 0.4 × 10^6^; chronic SCI: 2.7 ± 0.3 × 10^6^; *P* = 0.07) (Figure 5B). The expression of other exhaustion markers including CTLA-4, TIM-3 and LAG-3 on T-cells was not increased by chronic SCI (data not shown).

### Increased expression of PD1 ligand on B-cells and macrophages isolated from chronic SCI mice

**Figure 6:**
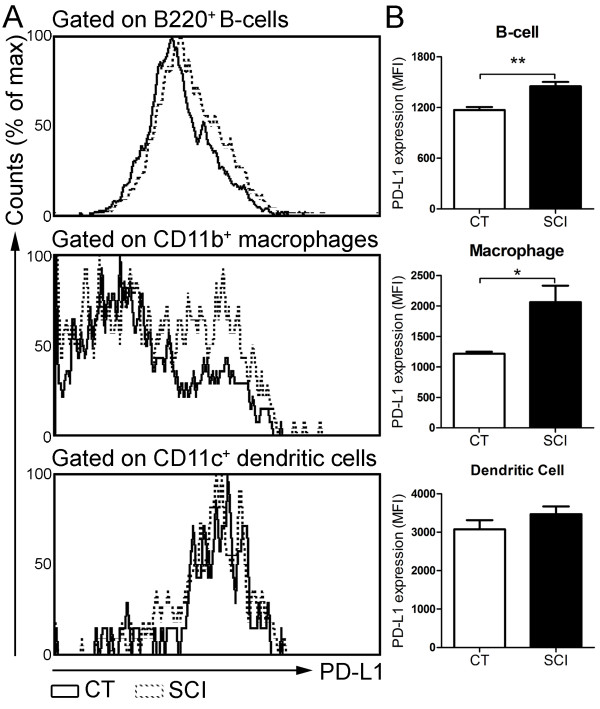
**Up regulation of PD-l ligand PD-L1 expression on splenic B-cells and macrophages following chronic spinal cord injury (SCI). (A)** Representative histogram plots show PD-L1 expression on gated B220^+^ B-cells, CD11b^+^ macrophages and CD11c^+^ dendritic cells from uninjured (CT) and T9-SCI mice at chronic phase after injury (SCI). **(B)** Bar graphs show the mean ± SEM of PD-L1 mean fluorescence intensities (MFI) in gated B-cells, macrophages and dendritic cells from CT and SCI mice. Ten thousand events gated on live singlets were collected. n = 4 mice per group. *P < 0.05, **P < 0.01, one-tailed Student’s *t*-test.

We sought to determine whether the ligand for PD-1 receptor (PD-L1) was up-regulated following chronic SCI, as engagement of PD-1 ligand with PD-1 induces T-cell exhaustion signaling. We measured the expression of PD-L1 on B-cells, macrophages and dendritic cells. Compared with CT group, the mean fluorescence intensity (MFI) values for PD-L1 were significantly increased on splenic B-cells (uninjured: 1,169 ± 38; chronic SCI: 1,451 ± 54; *P* = 0.003) and macrophages (uninjured: 1213 ± 34; chronic SCI: 2061 ± 270; *P* = 0.03) from the chronic SCI group (Figure 6A, B). However, PD-L1 expression on dendritic cells was similar between CT and SCI groups (uninjured: 3,074 ± 241; chronic SCI: 3,468 ± 202; *P* = 0.13) (Figure 6A, B).

### Blocking PD-1 restores cytokine production by CD8^+^ T-cells

**Figure 7:**
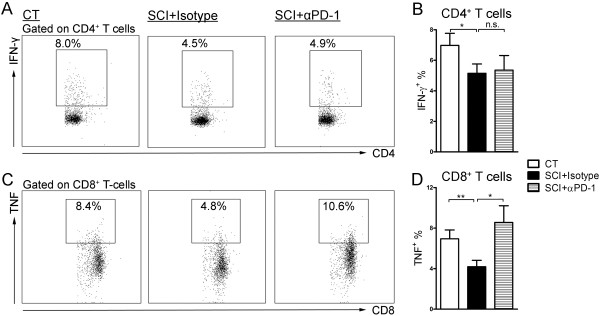
**Blocking PD-1 restores the TNF-α production by CD8**^**+ **^**T-cells from chronic spinal cord injury (SCI) mice.** Splenocytes (1 × 10^6^) isolated from uninjured mice were stimulated *ex vivo* with PMA/ionomycin in the presence of brefeldin A for four hours (group: CT). Splenocytes (1 × 10^6^) isolated from chronic SCI mice were also stimulated *ex vivo* with the same condition as the CT group, except that 10 μg/mL anti-PD-1 blocking antibody (group: SCI + αPD-1) or 10 μg/mL rat IgG2a, κ isotype (group: SCI + Isotype) were added to the culture. **(A)** Representative dot plots show the percentage of IFN-γ^+^ cells in gated CD4^+^ T-cells. **(B)** Bar graph represents the mean ± SEM percentage of IFN-γ^+^ cells in CD4^+^ T-cells. **(C)** Representative dot plots show the percentage of TNF-α^+^ cells in gated CD8^+^ T-cells. **(D)** Bar graph represents the mean ± SEM percentage of TNF-α^+^ cells in CD8^+^ T-cells. Ten thousand events gated on live singlets were collected. n = 9 for CT, n = 11 for SCI + Isotype, n = 11 for SCI + αPD-1. Data are pooled across three independent experiments. *P < 0.05, **P < 0.01, n.s. no significant difference was detected, *P* > 0.05, one-tailed Student’s *t*-test.

We next asked whether inhibiting PD-1 signaling will restore the functional defects in cytokine production observed in the splenic T-cells isolated from chronic SCI mice. Splenocytes from uninjured mice and chronic SCI mice were stimulated with PMA/ionomycin in the presence of either anti-PD-1 or an isotype control antibody. Compared with splenocytes from uninjured mice, the splenocytes from chronic SCI mice showed a significant reduction in the percentage of IFN-γ-expressing CD4^+^ T-cells (uninjured: 7.0 ± 0.8%; chronic SCI + Isotype: 5.1 ± 0.6%; *P* = 0.04) (Figure 7A, B) and TNF-α-expressing CD8^+^ T-cells (uninjured: 6.9 ± 0.9%; chronic SCI + Isotype: 4.2 ± 0.6%; *P* = 0.009) (Figure 7C, D) after PMA/ionomycin restimulation in presence of isotype control antibodies. Blocking PD-1 restored the percentage of CD8^+^ T-cells expressing TNF-α (chronic SCI + anti-PD-1: 8.6 ± 1.6%; *P* = 0.01) (Figure 7C, D). However, IFN-γ production by CD4^+^ T-cells from chronic SCI mice was not restored (chronic SCI + anti-PD-1: 5.4 ± 1.0%; *P* = 0.43) (Figure 7A, B).

### Chronic SCI increases PD-1 expression by altering sympathetic activity

**Figure 8:**
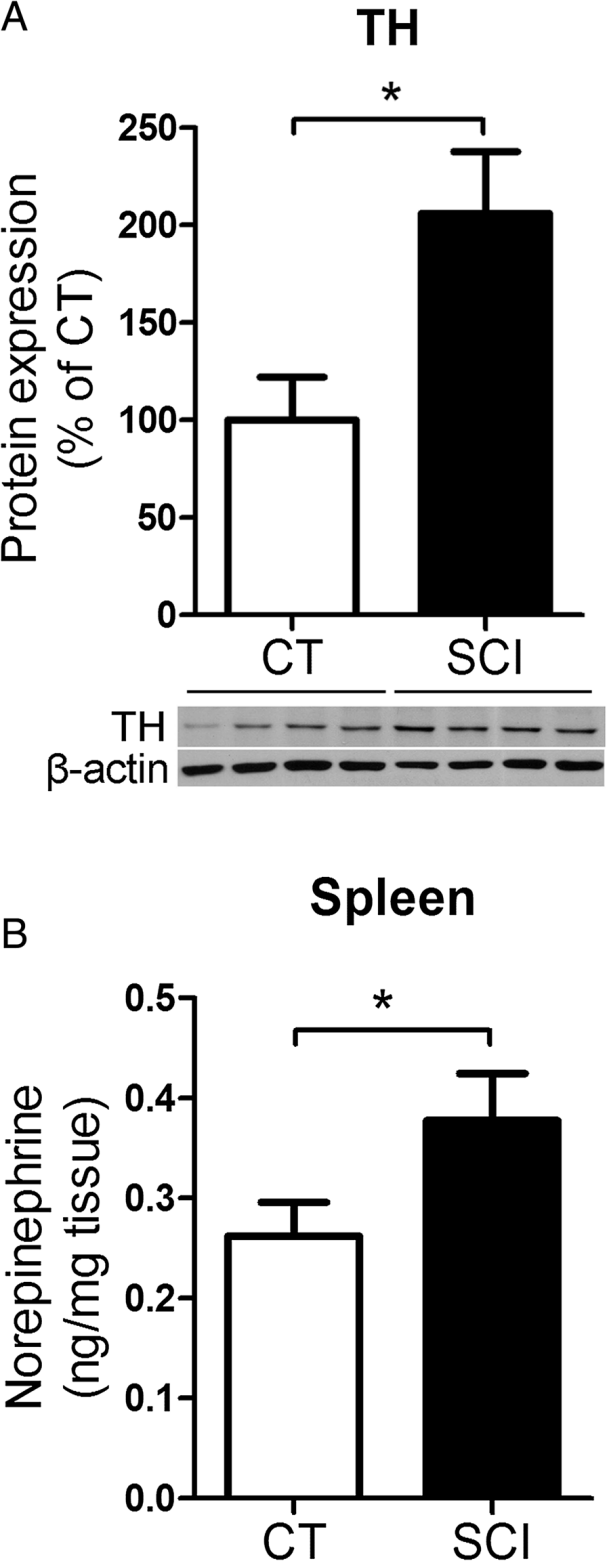
**Chronic spinal cord injury (SCI) increases the sympathetic activity in the spleen. (A)** Western blot quantification of tyrosine hydroxylase (TH) in the spleens of uninjured mice (CT) and chronic SCI mice (SCI). Data are normalized to the expression of β-actin. Bar graph represents the mean ± SEM of TH expression in the spleen protein extract and are expressed as percentage of CT. n = 4 mice/group. Data represents two independent experiments. *P < 0.05, two-tailed Student’s *t*-test. **(B)** Bar graph represents the mean ± SEM of NE concentration (ng/mg) in the spleen homogenates from CT and SCI mice. n = 5 mice/group. *P < 0.05, one-tailed Student’s *t*-test.

**Figure 9:**
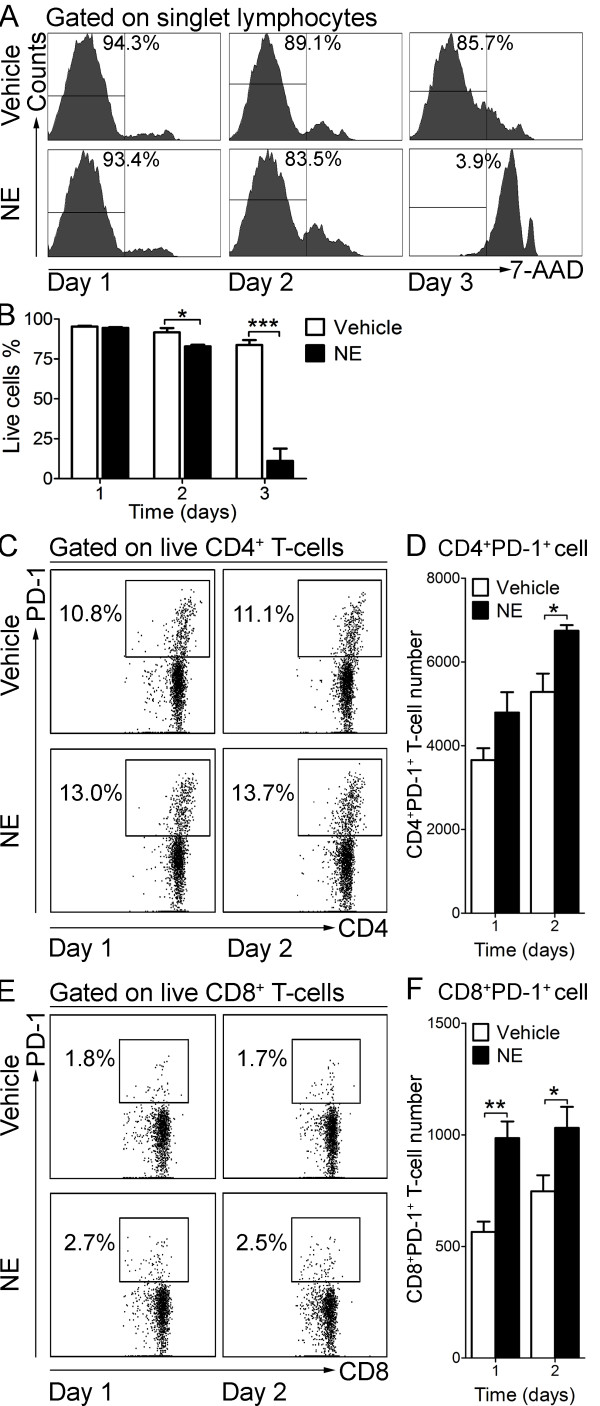
**Norepinephrine (NE) up-regulates PD-1 expression on T-cells *in vitro*.** Enriched splenic T-cells (10^6^ cells/ml) were stimulated with 10 μM (NE) or its vehicle (Vehicle) *in vitro* for one to three days. Cells were collected and processed for flow cytometry. **(A)** Representative histograms show the 7-AAD fluorescence and the percentage of live cells (7-AAD^−^) in gated singlet lymphocytes. **(B)** Bar graph shows the mean ± SEM percentage of live cells (7-AAD^−^) following one to three days of *in vitro* culture with NE or vehicle. Experiments were performed in triplicate. *P < 0.05, ***P < 0.001, two-tailed Student’s *t*-test. **(C)** Representative dot plots show the percentage of PD-1^+^ cells in gated live CD4^+^ T-cells. **(D)** Bar graph represents the mean ± SEM number of CD4^+^ PD-1^+^ live cells per well after one and two days of NE or vehicle treatment. **(E)** Representative dot plots show the percentage of PD-1^+^ cells in gated live CD8^+^ T-cells. **(F)** Bar graph represents the mean ± SEM number of live CD8^+^PD-1^+^ cells per well after one and two days of NE or vehicle treatment. Ten thousand events gated on singlet lymphocytes were collected. Experiments were performed in triplicate. *P < 0.05, **P < 0.01, one-tailed Student’s *t*-test.

To understand how chronic SCI induces T-cell exhaustion, we investigated whether the activity of the sympathetic nervous system is altered by SCI. As a surrogate marker of SNS activity we measured the protein expression level of tyrosine hydroxylase (TH), the rate-limiting enzyme for catecholamine (CA) synthesis, in the spleen extract from both control and SCI mice. Compared to uninjured control, TH expression levels in the spleen of injured mice was significantly elevated (Figure 8A) and consistent with significantly higher NE concentrations in the spleen of those injured mice (uninjured: 0.26 ± 0.03 ng NE/mg spleen; chronic SCI: 0.38 ± 0.05 ng NE/mg spleen, *P* = 0.04) (Figure 8B). These results led us to hypothesize that higher sustained splenic NE levels in injured mice may contribute to T-cell exhaustion. To test this hypothesis, we sought to incubate enriched T-cells from naive mice with 10 μM NE over several days and assess whether these cells up-regulated PD-1. Since prolong exposure to NE could be toxic to the cells, we first determined cell viability. Naïve enriched T-cells were incubated either with NE or vehicle for one, two and three days and cell viability was measured by flow cytometry using 7-AAD. As shown in Figure 9, after three days in culture with NE, T-cell viability was very low, as reflected by a low percentage of 7-AAD^−^ live cells (Vehicle: 83.7 ± 3.1%; NE: 11.1 ± 7.7%; *P* = 0.0009) (Figure 9A, B). Therefore, we determined the number of PD-1 expressing T-cell following one and two days of NE stimulation. As shown in Figure 9C-F, one day of NE stimulation significantly increased PD-1^+^CD8^+^ T-cell number compared to vehicle-treated cells (Vehicle: 565 ± 46; NE: 985 ± 75; *P* = 0.004), when the cell numbers of both CD4^+^PD-1^+^ T-cells (Vehicle: 5,283 ± 439; NE: 6,747 ± 135; *P* = 0.02) and CD8^+^PD-1^+^ T-cells (Vehicle: 747 ± 72; NE: 1,031 ± 94; *P* = 0.04) were upregulated after two days of continuous NE stimulation.

**Figure 10:**
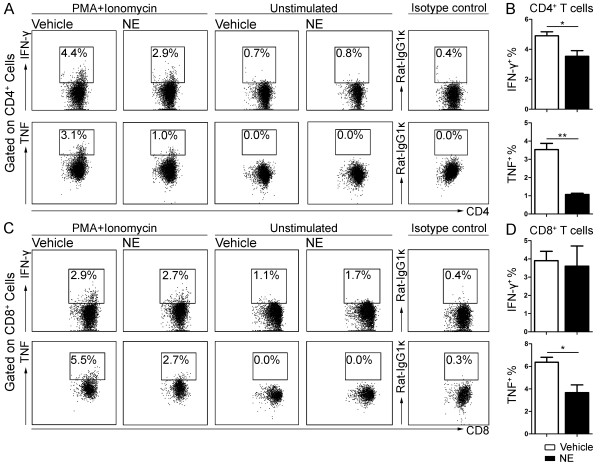
**Exposure to norepinephrine (NE) *in vitro *impairs T-cell cytokine production in response to PMA/ionomycin stimulation.** Enriched splenic T-cells (10^6^ cells/ml) were cultured with 10 μM NE or its vehicle (Vehicle) *in vitro*. After two days of NE exposure, cells were stimulated with PMA/ionomycin for four hours in the presence of brefeldin A. Intracellular cytokine staining and flow cytometry analysis were performed to measure cytokine production. **(A)** Representative dot plots show the percentage of IFN-γ^+^ cells and TNF-α^+^ cells in gated CD4^+^ T-cells following PMA/ionomycin stimulation or with brefeldin A only (unstimulated). **(B)** Bar graph represents the mean ± SEM percentages cytokine producing CD4^+^ T-cell in response to PMA/ionomycin stimulation. **(C)** Representative dot plots show the percentage of IFN-γ^+^ cells and TNF-α^+^ cells in gated CD8^+^ T-cells following PMA/ionomycin stimulation or with brefeldin A only (unstimulated). **(D)** Bar graph represents the mean ± SEM percentages of cytokine producing CD8^+^ T-cells in response to PMA/ionomycin stimulation. Ten thousand events gated on lymphocytes were collected. Experiments were performed in triplicate, *P < 0.05, **P < 0.01, one-tailed Student’s *t*-test.

We next determined whether NE stimulation resulted in defective cytokine production in the enriched T-cells. Enriched T-cells were incubated for two days with NE prior to adding PMA/ionomycin and intracellular cytokine profile of these cells was characterized after 4 hours of stimulation. Compared with vehicle control, NE stimulation significantly reduced both IFN-γ (Vehicle: 4.9 ± 0.3%; NE: 3.5 ± 0.4%; *P* = 0.02) and TNF-α production (Vehicle: 3.5 ± 0.3%; NE: 1.1 ± 0.1%; *P* = 0.001) of CD4^+^ T-cells (Figure 10A, B), as well TNF-α production (Vehicle: 6.4 ± 0.4%; NE: 3.7 ± 0.7%; *P* = 0.01) in CD8^+^ T-cells (Figure 10C, D), These data strongly suggest that sustained elevated levels of splenic NE following chronic SCI could induce T-cell exhaustion and dysfunction of cytokine production.

## Discussion

In the present work, we assessed the impact of chronic SCI on the peripheral immune system and explored the mechanisms by which traumatic injury to the spinal cord induces T-cell dysfunction. Using a severe spinal cord contusion model at thoracic level T9, we demonstrate that although there was no change in splenic T-cell numbers after five weeks following injury, their function was significantly altered as assessed by *ex vivo* stimulation with PMA/ionomycin or with anti-CD3/anti-CD28 stimulation. IFN-γ production by CD4^+^ T-cells and TNF-α production by CD8^+^ T-cells in response to PMA/ionomycin stimulation were significantly reduced in the cells isolated from chronically spinal cord injured mice. Chronic SCI impaired the IFN-γ and TNF-α production of both CD4^+^ and CD8^+^ T-cells following anti-CD3/anti-CD28 stimulation. We provide evidence that T-cell exhaustion contributes to SCI-induced T-cell dysfunction. T-cell expression levels of exhaustion marker PD-1 was significantly increased by chronic SCI, while *in vitro* blockade of PD-1 restored CD8^+^ T-cell function. To our knowledge, this is the first report showing that chronic SCI alters T-cell function and increases T-cell exhaustion. Furthermore, the activity of SNS in the spleen is higher in injured mice. Long-term *in vitro* exposure to NE increased PD-1 expressing T-cells and impaired T-cell function of cytokine production. This finding suggests that alterations in the SNS output is involved in the mechanism by which chronic SCI induces T-cell exhaustion, and highlights the importance of the SNS in the regulation of T-cell function.

Previous studies in SCI animal models have characterized changes in the peripheral immune system occurring in the acute phase. T-cell loss in the spleen was reported at one to three days after injury [31,32]. Increased level of glucocorticoids and NE at acute phase after SCI induces lymphocyte apoptosis and results in T-cell decrease [41]. However, no significant reduction in the T-cell number was observed at later time points such as day 7, day 14 and day 28 post-injury [16,31]. Consistent with those studies, we did not find any significant difference in the number of either CD4^+^ or CD8^+^ T-cell between uninjured and chronic SCI mice (> five weeks post-injury). These results indicate that the temporary T-cell loss in the acute phase after SCI does not persist in the chronic phase. Therefore the SCI-induce immune depression in the chronic phase is more likely due to functional defects of immune cells rather than their number.

T-cells regulate host immunity against pathogen infection by secreting cytokines upon activation [36]. We first used *ex vivo* PMA/ionomycin stimulation to investigate the effect of chronic SCI on T-cell cytokine production. In this study, chronic SCI mice showed a reduction in IFN-γ production by CD4^+^ T-cells as well as TNF-α production by CD8^+^ T-cells. We also measured the T-cell production upon stimulation with anti-CD3/anti-CD28, which is more relevant to physiological T-cell receptor activation *in vivo*. We demonstrated that the cytokine production by both CD4^+^ T-cells and CD8^+^ T-cells are impaired. These defects could be associated with SCI-induced immune depression. IFN-γ is a crucial modulator in multiple immune responses, including macrophage activation, major histocompatibility complex (MHC) I and MHC II antigen presentation up-regulation, lymphocyte recruitment, CD4^+^ T-helper response and inhibition of viral replication. The deregulation of IFN-γ production may contribute to the impaired capacity to combat infection in chronic SCI patients [33]. TNF-α plays a key role in both host inflammatory and cytotoxic responses against pathogens [42]. Specifically, during viral infection, TNF-α produced by cytotoxic T-cells is responsible for apoptosis and lysis of virus-infected cells [34,35]. Therefore, insufficient TNF-α production by T-cells is a potential cause of SCI-induced immunodeficiency. Interestingly, some studies have suggested a role for TNF-α in forming the germinal center and generating humoral responses [43-45]. Alteration in TNF-α production by T-cell in injured mice may contribute to their defects in mounting an appropriate antibody response.

T-cell exhaustion has been well studied in the last decade for its role in T-cell dysfunction and immunodeficiency. It was first described in a lymphocytic choriomeningitis virus (LCMV) chronic infection mouse model as a virus-specific CD8^+^ T-cell population which cannot elaborate efficient antiviral effectors’ function [17]. Since then, T-cell exhaustion has been investigated in many chronic viral infections including human immunodeficiency virus (HIV), hepatitis B virus and hepatitis C virus, and cancer as well as aging models [18-23,25,46]. To our knowledge, our study is the first to show T-cell exhaustion in a SCI model. Previous studies have demonstrated a correlation between higher PD-1 expression and reduced cytokine production in CD8^+^ T-cells [18,24,26]. Herein, we showed that chronic SCI mice have a significantly higher number of CD8^+^ T-cells expressing PD-1 along with increased expression of PD-L1, the ligand for PD-1, on B-cells and macrophages. Moreover, the production of TNF-α by CD8^+^ T-cells was restored *in vitro* by blocking PD-1 signaling. These results suggest that higher PD-1 expression contributes to SCI-induced CD8^+^ T-cell dysfunction. However, the number of CD4^+^ T-cells expressing PD-1 was not significantly up-regulated by chronic SCI and blocking PD-1 failed to restore IFN-γ production by CD4^+^ T-cells suggesting that other mechanisms are involved in SCI-induced CD4^+^ T-cell dysfunction.

We next explored how chronic SCI increases PD-1 expression on CD8^+^ T-cells. Since the spleen is innervated and modulated by the SNS, disruption of the sympathetic preganglionic neurons at the injury level may lead to altered SNS output to the spleen. Post-acute phase SCI causes reorganization of synapses on the sympathetic preganglionic neurons and reinnervation of the sympathetic terminals at the target organs [47-49]. Our results showed higher levels of TH in the splenic protein extract from injured animals compared to uninjured controls. As the rate-limiting enzyme for catecholamine synthesis, TH has been used to identify catecholamine containing noradrenergic terminals in the spleen [50,51]. Elevated TH levels in the spleen of chronic SCI mice correlated with higher splenic NE levels. These data could be explained by increased sympathetic innervation, higher catecholamine levels per cell or more endogenous catecholamine produced by lymphocytes in the spleen [52,53]. NE has been reported to regulate the functions of immune cells [10]. Particularly, experimental induction of autonomic dysreflexia in chronic SCI mice causes splenic NE accumulation, which is involved in the impaired immune function [14]. We hypothesized that higher NE levels in the spleen following chronic SCI were responsible for the increased T-cell exhaustion. Consistent with this hypothesis, we found that the number of PD-1 expressing T-cells was increased after prolonged exposure to NE *in vitro*. While it is unclear how NE regulates PD-1 expression, several transcription factor pathways have been demonstrated to play a role in regulating T-cell exhaustion. Specifically, B-lymphocyte-induced maturation protein 1 (Blimp-1), nuclear factor of activated T-cells cytoplasmic 1 (NFATc1) and Notch signaling are regulators of PD-1 gene expression, whereas high expression of T-bet suppresses PD-1 expression [54-57]. There is no direct evidence that NE regulates these transcription pathways in murine splenic T-cells. However, NE was reported to increase NFATc1 activity in primary neonatal cardiomyocyte culture [58], suggesting that NE stimulation may up-regulate PD-1 expression by activating NFATc1. Interestingly, NE stimulation of T-lineage cells increases the phosphorylation of phosphor 38 mitogen-activated protein kinase (p38 MAPK), which is involved in the mechanisms by which HIV-1 Nef protein induces PD-1 expression [59,60]. The activation of the p38 MAPK pathway may also contribute to the higher PD-1 expression in our model. Prolonged NE exposure resulted in decreased T-cell cytokine production. Consistent with our findings, several research groups have found that activation of β2 adrenergic receptor inhibits IFN-γ production in T-cells [61,62]. As NE can affect T-cell function by multiple mechanisms, such as increased intracellular cAMP levels [63], we suggest that higher expression of exhaustion marker PD-1 on CD8^+^ T-cells participates in the mechanisms of NE-induced T-cell dysfunction.

## Conclusions

In conclusion, we demonstrated that chronic SCI impaired both CD4^+^ and CD8^+^ T-cell functions. Furthermore, we identified T-cell exhaustion as a possible mechanism by which chronic SCI leads to impaired CD8^+^ T-cell function. Alterations in the SNS contributed to the exhausted phenotype of CD8^+^ T-cells from injured mice. These findings highlight the role of the nervous system and neurotransmitters in regulating peripheral immunity. Our study also sheds light on the development of therapeutic strategies to reduce re-hospitalization and death rate from infection in chronic SCI patients. For example, clinical trials using antagonist antibodies to PD-1 are now ongoing for cancer treatment [64]. Our research provides evidence for the clinical application of PD-1 antibodies in SCI-induced immune depression treatment. In addition, adrenergic receptor antagonists, which have been widely used in cardiovascular diseases, constitute also a potential treatment to restore immunity in chronic SCI patients.

## Abbreviations

BSA: bovine serum albumin; CA: catecholamine; CFSE: carboxyfluorescein diacetate succinimidyl ester; CT: uninjured mice; CTLA-4: cytotoxic T-lymphocyte antigen 4; ELISA: enzyme-linked immunosorbent assay; FACS: flow cytometry; FBS: fetal bovine serum; HBSS: Hank’s Balanced Salt Solution; HIV: human immunodeficiency virus; IFN-γ: interferon gamma; LAG-3: lymphocyte activation gene-3; MFI: mean fluorescence intensity; MHC: major histocompatibility complex; NE: norepinephrine; NFATc1: nuclear factor of activated T-cells cytoplasmic 1; p38 MAPK: phosphor 38 mitogen-activated protein kinase; PD-1: programmed cell death-1; PCA: perchloric acid; PMA: phorbol myristate acetate; SDS-PAGE: sodium dodecyl sulfate polyacrylamide gel electrophores; SEM: standard error of the mean; SCI: spinal cord injury; SNS: sympathetic nervous system; T9: thoracic level 9; TBST: Tris-buffered saline with Tween-20; TCR: T-cell receptor; TH: tyrosine hydroxylase; TIM-3: T-cell immunoglobulin mucin-3; TNF: tumor necrosis factor.

## Competing interests

The authors declare that they have no competing interests.

## Authors’ contributions

JZ participated in the study design, performed the experiments, analyzed the data and wrote the manuscript. AS and SA performed the T-cell proliferation assay and analyzed the data. VBR participated in the study design and the animal experiments, performed Western blot experiments and wrote the manuscript. JRB conceived the study and helped draft the manuscript. All authors read and approved the final manuscript.

## Supplementary Material

Additional file 1 **Gating strategy and isotype controls for the identification of CD4**^**+**^** T-cells and CD8**^**+ **^**T-cells.** Total cells were gated based on forward scatter (FSC) and side scatter (SSC). Doublets were excluded using FSC width versus area, followed by SSC width versus area. T-cells were gated as CD45^+^CD3^+^ cells. CD4^+^ T-cells and CD8^+^ T-cells were gated based on the expression of CD4 and CD8 with their isotype controls. Click here for file

Additional file 2 **Gating strategy and isotype controls for the identification of cytokine producing T-cells.** Total cells were gated based on forward scatter (FSC) and side scatter (SSC). Doublets were excluded using FSC width versus area, followed by SSC width versus area. CD4^+^ T-cells and CD8^+^ T-cells were gated based on the expression of CD4 and CD8. The expression of IFN-γ and TNF-α on CD4^+^ T-cells and CD8^+^ T-cells was gated using their isotype controls. Click here for file
