# Supplementary figures and images for: Chronic thoracic spinal cord injury impairs CD8+ T-cell function by up-regulating programmed cell death-1 expression
Source: J Neuroinflammation. 2014 Apr 1;11:65. doi: 10.1186/1742-2094-11-65 (PMC4230802; doi:10.1186/1742-2094-11-65)

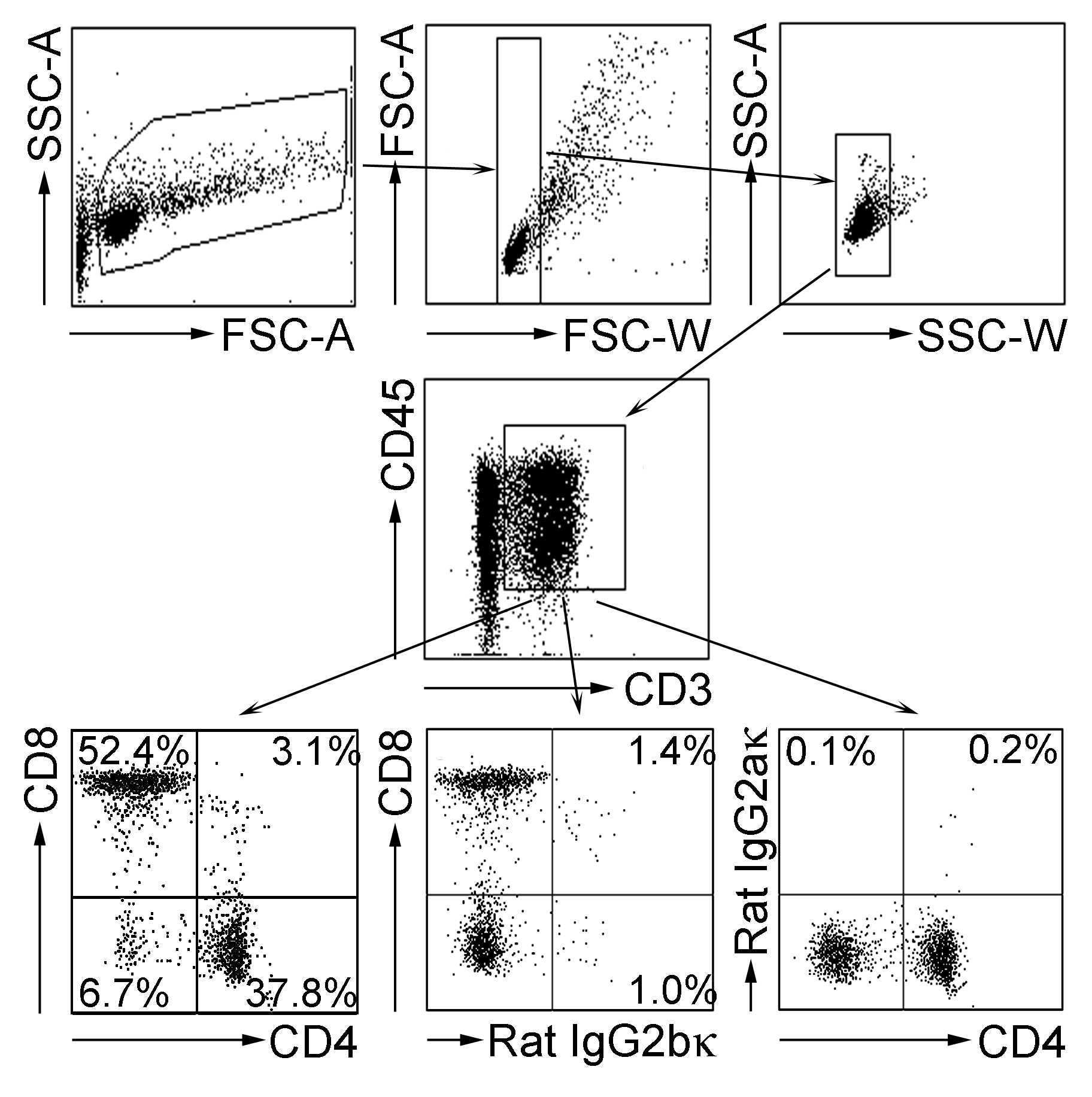

Supplement: Additional file 1 — Gating strategy and isotype controls for the identification of CD4+ T-cells and CD8+ T-cells. Total cells were gated based on forward scatter (FSC) and side scatter (SSC). Doublets were excluded using FSC width versus area, followed by SSC width versus area. T-cells were gated as CD45+CD3+ cells. CD4+ T-cells and CD8+ T-cells were gated based on the expression of CD4 and CD8 with their isotype controls. [file 1742-2094-11-65-S1.tiff]

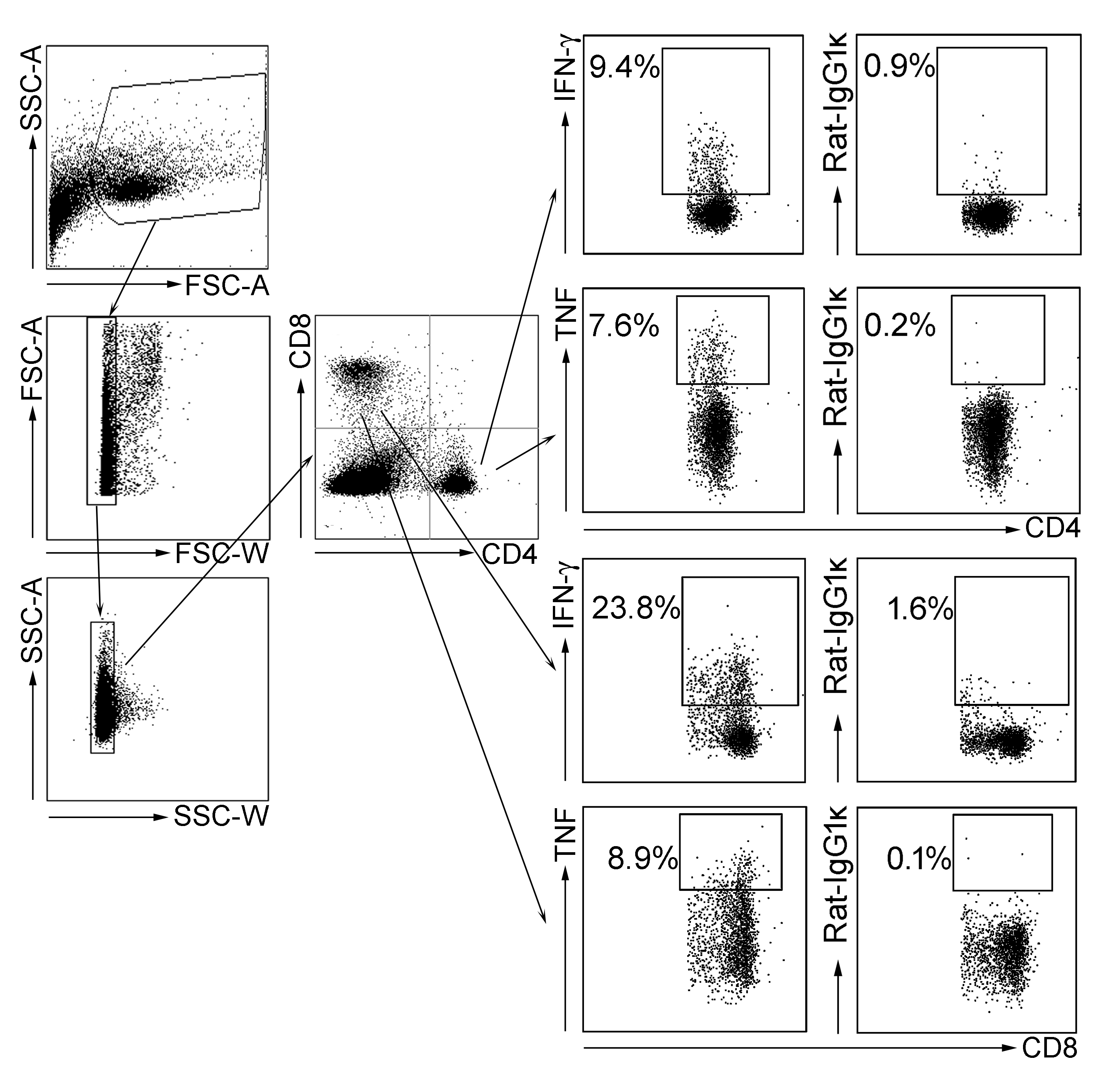

Supplement: Additional file 2 — Gating strategy and isotype controls for the identification of cytokine producing T-cells. Total cells were gated based on forward scatter (FSC) and side scatter (SSC). Doublets were excluded using FSC width versus area, followed by SSC width versus area. CD4+ T-cells and CD8+ T-cells were gated based on the expression of CD4 and CD8. The expression of IFN-γ and TNF-α on CD4+ T-cells and CD8+ T-cells was gated using their isotype controls. [file 1742-2094-11-65-S2.tiff]
